# 

**Published:** 1913-10-11

**Authors:** 


### A Chaplain on Hospital Work.

Collections were made for the hospitals at the Bovey Tracey harvest festival services in the parish church on Sunday, October 5. The Rev. S. B. Taylor, M.A., chaplain (retired) of H.M. Bengal Establishment, who was the preacher in the morning, paid tribute in general to the self-sacrifice of doctors and hospital nurses, and then stated how on visiting the Bristol Royal Infirmary the previous week he had noted in the chapel certain tablets to the memories of various doctors who had met death from blood-poisoning and other causes contracted during their work. He also saw one to the memory of a nurse, Elizabeth Croft, who was but twenty-five when she passed away. In fact, all these courageous personages were under forty years of age. The governing body of the Bristol Royal Infirmary gave assurance that whenever medical or surgical or nursing appointments were vacant the applications were plentiful. This was an indication that the enthusiasm for engaging in a beneficent but dangerous profession was widespread.

### THE NAVAL MEDICAL SERVICE.

At the examination for appointments as acting surgeons, Naval Medical Service, held on September 29 and October 1 and 2, thirteen candidates were successful and obtained the following marks :—

| Name | Marks | Medical School |
|---|---|---|
| J. S. Elliot, M.B. ... | 1,703 | Edinb. Univ. |
| A. J. Tonkinson, M.B. | 1,686 | London Hosp. |
| A. R. Sharrod, M.B. ... | 1,643 | London Hosp. |
| G. J. Kilbride, M.B. ... | 1,578 | Cecilia Street Medical School, Dublin |
| A. E. Panter, B.A. ... | 1,515 | Camb. Univ. and King's Coll. Hosp., London |
| G. S. Harvey, M.B. ... | 1,483 | Univ. Coll., Cork |
| J. P. Blockley, M.B. ... | 1,470 | Edinb. Univ. |
| W. H. Murray, M.B. ... | 1,455 | Univ. Coll., C rk |
| J. R. Haldane, M.B. ... | 1,455 | Glasgow Univ. |
| G. H. Hayes, M.B. ... | 1,435 | Univ. Coll., Cork |
| W. S. Birch ... ... | 1,418 | King's Coll. Hosp. |
| H. C. A. T. Cannon ... | 1,380 | Royal Coll. of Surgeons, Ireland |
| H. J. Hopps, M.B. ... | 1,338 | Edinb. Univ. |

The maximum number of marks obtainable is 2,400.

